# Design of Chopsticks-Shaped Heating Resistors for a Thermal Inkjet: Based on TaN Film

**DOI:** 10.3390/mi13050787

**Published:** 2022-05-18

**Authors:** Anjiang Lu, Xishun Peng, Qiliang Sun, Jin Cheng, Naitao Xu, Yibo Xie, Jie Ding, Pangyue Li, Ji’an Long, Jiawen Wu

**Affiliations:** 1 College of Big Data and Information Engineering, Guizhou University, Guiyang 550000, China; ajlu@gzu.edu.cn (A.L.); dingjie_0618@163.com (J.D.); lja19990322@163.com (J.L.); 2 Wuxi V-Sensor Technology Co., Ltd., Wuxi 214000, China; abc810801@126.com (Q.S.); wuaven@outlook.com (J.W.); 3 School of Optoelectronic Engineering, Xi’an Technological University, Xi’an 710000, China; 624788236@163.com (J.C.); xunaitao_123@163.com (N.X.); 13319215096@163.com (Y.X.); lipangyue971230@163.com (P.L.)

**Keywords:** MEMS, thermal inkjet, TaN chopsticks-shaped, Si_3_N_4_ film, SiO_2_ film

## Abstract

Efficient printing frequency is critical for thermal bubble inkjet printing, while the difficulty lies in the structural design and material selection of the heating resistors. In this paper, a TaN film was used as the main material of the heating resistors, and two TaN films were placed in parallel to form the chopsticks-shaped structure. The heating time was divided into two sections, in which 0–0.1 μs was the preheating and 1.2–1.8 μs was the primary heating. At 1.8 μs, the maximum temperature of the Si_3_N_4_ film could reach about 1100 °C. At the same time, the SiO_2_ film was added between the TaN film and Si_3_N_4_ film as a buffer layer, which effectively avoided the rupture of the Si_3_N_4_ film due to excessive thermal stress. Inside the inkjet print head, the maximum temperature of the chamber reached about 680 °C at 2.5 μs. Due to the high power of the heating resistors, the working time was greatly reduced and the frequency of the inkjet printing was effectively increased. At the interface between the back of the chip and the cartridge, the SiO_2_ film was used to connect to ensure a timely ink supply. Under the condition of 12 V at 40 kHz, the inkjet chip could print efficiently with 10 nozzles at the same time. The inkjet chip proposed in this paper is not limited to only office printing, but also provides a new reference for 3D printing, cell printing, and vegetable and fruit printing.

## 1. Introduction

As microfluidic chips become significant equipment in our daily lives, such as in office printing, 3D printing, cell bioprinting, and food printing, the sizes and structures of inkjet chips are constantly being upgraded [1,2]. In sensor manufacturing, screen printing [3] and inkjet printing [4,5] have a high penetration rate. In addition, there are 3D printing, flexographic printing [6], laser printing [7], and other new fields. In recent years, food printing has received more attention. Since researchers at Cornell University used the technology of extrusion printing to make food in 2007, the market for food printing has gradually expanded. Up to now, the mainly technology for food printing included hot melt extrusion (HME), binder jetting (BJ), and inkjet printing. Due to the high-performance requirements of inkjet chips, the HME and BJ methods are used more frequently in food printing. Researchers had already studied the adaptation of different foods in 3D printing, such as chocolate 3D printing [8], cheese printing [9], vegetable printing, and fruit printing [10,11,12]. The market more obviously reflects vegetable and fruit printing because people are seeking healthy food to replace junk food. The World Health Organization recommends that people eat more than 400 g of fruits and vegetables daily, which strongly pushes the technology of vegetable and fruit printing forward. Yang Fanli et al. [12] researched the influence of mixed samples of lemon juice and potato starch at different proportions on printing performance. Azam et al. [13] researched a mixture of wheat starch and orange juice as a printing material.

**Figure 1:**
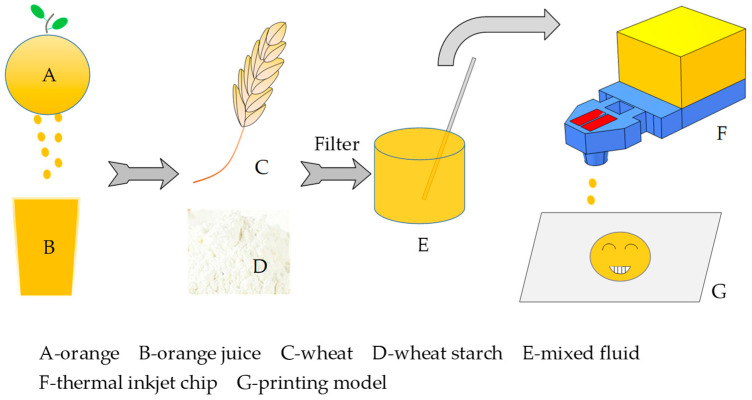
Orange juice and wheat starch were used in inkjet printing.

Among printing devices, a thermal inkjet uses the instantaneous heating of heating resistors to form a part of the microfluid in the chamber to be vaporized and nucleate a vapor bubble, and the high pressure generated by this process causes the microfluid to be ejected from the chamber. Generally, the printing frequency of a piezoelectric inkjet (up to 30 kHz) [14,15] is higher than that of a thermal inkjet, which is critical for vegetable and fruit printing. However, for the piezoelectric inkjet chip, its sealing requirements are extremely strict [16], and the antifatigue ability of the piezoelectric vibration plate is also a key factor in its high cost. In addition, the piezoelectric chip is installed in the printer, which is difficult to disassemble. To pave the path for improving the performance of inkjet printing in the field of food printing, our team designed a thermal inkjet chip with a high printing frequency of 40 kHz. Because the mixed fluid contained wheat starch, it easily solidified after ejection, and a slow printing frequency would affect the appearance of the molding. In addition, a slow printing frequency can cause a clogging phenomenon inside the chip, and wheat starch in the mixed fluid easily forms sediment in the chamber. The inkjet chip designed in this paper had a printing frequency as high as 40 kHz. Due to the high-power density of the heating resistors, the explosive pressure generated in the chamber was enough to eject the fluid in a short time, which avoided the inkjet quality degradation caused by an insufficient pressure. As shown in Figure 1, the TaN chopsticks-shaped heating resistors of the thermal inkjet chip provided a new choice for fruit and vegetable printing equipment. Firstly, the orange was squeezed into juice. We mixed the juice with the wheat starch and used the filter paper to remove the large particles. Then, the viscosity-measuring instrument was used to adjust the liquid viscosity. Finally, we introduced the mixture into the ink cartridge for inkjet printing.

The TaN chopsticks-shaped heating resistors were mainly fabricated with a TaN film [17], using two parallel TaN films to form the chopsticks-shaped structure, which caused the microfluid to instantly vaporize in the chamber. The TaN film had a high-power density, which reduced the heating time and improved the printing frequency. In addition, the TaN film had good resistance to acid and moisture, with a melting temperature of 3090 °C and strong stability [18]. The SU-8 photoresist had excellent thermal and chemical stability [19,20], and was used to fabricate the chamber and nozzle. The top diameter of the nozzle was about 18 μm, which ensured the accuracy of the model appearance.

## 2. Actuation Concept and Simulation

### 2.1. Establishment of Theoretical Model

In this study, we proposed to use the TaN film as the basic material of the heating resistors in the thermal inkjet chip. The conception of Fourier’s law needed to be considered before designing the chip [21]. According to the definition of Fourier’s law, in thermal conduction, in the vertical direction of the given section, the thermal transfer through the section per unit time is proportional to the rate of temperature variation and the area of the section, while the direction of the thermal transfer is opposite to the direction of temperature rise:

(1) $$J_{T}=-k\frac{dT}{dz}$$

where $J_{T}$(W/m^2^) is the heat flux rate at the *z* direction of the section, $\frac{dT}{dz}$ is the temperature gradient at the *z* direction, and *k* is the coefficient of thermal conductivity. We needed to pay attention to the minus sign, which indicates that the direction of thermal transfer is opposite to the direction of the temperature gradient.

In order to research the phenomenon of thermal conduction, we needed to relate the voltage, current, and other factors to the thermal transfer. The coefficient of thermal conductivity *k* can be inferred as:

(2) $$k=\frac{Q}{t}\frac{L}{A\nabla T}$$

where *Q* is the amount of heat of the resistor, *t* is the heating time, *L* is the length of the heating resistors, *A* is the area of the heating resistors, and ∇T is the temperature difference between adjacent objects. The heat *Q* can be inferred as:

(3) $$Q=I^{2}Rt$$

where *I* is the electric current, *R* is the value of the resistors, and *t* is the heating time. Therefore, the formula of thermal conduction in the *z* direction can be inferred as:

(4) $$\frac{\partial }{\partial z}\left(k\frac{\partial T}{\partial z}\right)+\frac{E^{2}}{RV}=\rho c\frac{\partial T}{\partial t}$$

where *E* is the driving voltage, *V* is the volume of resistors, ρ is the density, and *c* is the value of specific heat capacity. In three-dimensional space, the temperature varies with time and spatial coordinates, along with the thermal generation and consumption. At this time, Fourier’s law in three-dimensional space is:

(5) $$J_{T}=-k\nabla T=-k\left(\vec{i}\frac{\partial T}{\partial x}+\vec{j}\frac{\partial T}{\partial y}+\vec{z}\frac{\partial T}{\partial z}\right)$$

**Figure 2:**
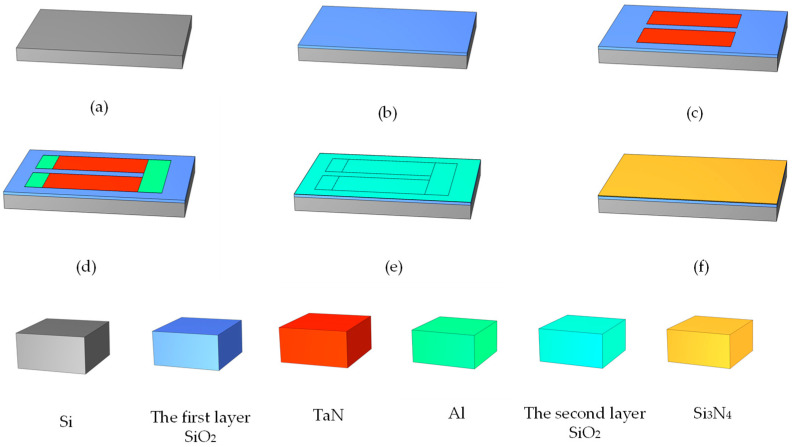
The fabrication of the TaN chopsticks-shaped heating resistors. (**a**) Using bare silicon as the base. (**b**) Deposition of the first layer of SiO_2_. (**c**) Deposition of heating resistors. (**d**) Aluminum as electrode. (**e**) Deposition of the second layer of SiO_2_. (**f**) Deposition of Si3N4 film.

As shown in Figure 2, we used the bare silicon as the base of the heating resistors, and its surface was covered with a layer of SiO_2_ [22]. When an electric current was passed through the TaN film, since the Si was a semiconductor material, the current would flow to it, so SiO_2_ was used as an insulating layer to prevent the current from flowing into the Si [23]. The TaN film, as a chemical material, is usually used to fabricate accurate heating resistors, as it can resist water vapor erosion, which is significant in inkjet printing. The two ends of TaN film were connected with Al, and the electric current entered from the right side and flowed out to the left side. The area flowing through the TaN was the core area of the heating resistors. Then, the second layer of SiO_2_ was covered on the surface of TaN film; because the Si_3_N_4_ was a structural ceramic [24,25], its coefficient of thermal expansion was about 2.3×10^−6^/K, and its coefficient of thermal conductivity was about 20(W/(m·K)), while the thermal conductivity of the TaN was about 9.54(W/(m·K)). If the two sections contacted directly, the Si_3_N_4_ was prone to rupturing due to the excessive thermal stress under instantaneous electric heating [26], so we used the SiO_2_ as a buffer layer to play a balancing role. The simulation parameters of the TaN chopsticks-shaped heating resistors are shown in Table 1.

### 2.2. Simulation

The COMSOL finite element software was used to simulate the working situation of the heating resistors. We needed to mesh the 3D model of the TaN chopsticks-shaped heating resistors.

**Figure 3:**
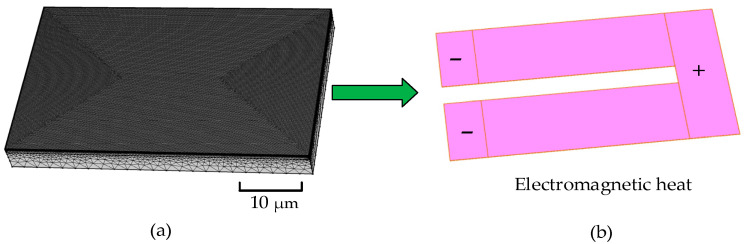
The physical field model of the TaN chopsticks-shaped heating resistors: (**a**) meshed 3D model of heating resistors; (**b**) electromagnetic heating model of the TaN film.

As shown in Figure 3a, the 3D model was divided into 1,481,025 grid cells. The physical field we chose was the electromagnetic heating and solid thermal transfer [27]. Since the chamber was above the heating resistors, the fluid heat dissipation was set as the heat-flux condition at the top of the heating resistors. In addition, in order to simplify the complexity of the 3D model and simulate the heating process as realistically as possible, we finally set the Si to 5 μm by analyzing the thermal conductivity coefficient of the material, while the actual condition was about 300 μm. The reason was that the instantaneous heating time was too short for the temperature to diffuse out, and we were more concerned with the temperature of the cross section that directly contacted the microfluid.

In order to research the working condition of the heating resistors under the different voltages, we set the voltages at 6 V, 8 V, 10 V, 12 V, and 14 V. As shown in Figure 3b, the chopsticks-shaped heating resistors shared the same electric current inlet. After the currents flowed through the TaN film, they were grounded and exported. During the entire simulation process, the temperature of the Si_3_N_4_ film was significant because the Si_3_N_4_ film contacted the chamber directly. Table 2 lists the main parameters of the materials.

**Figure 4:**
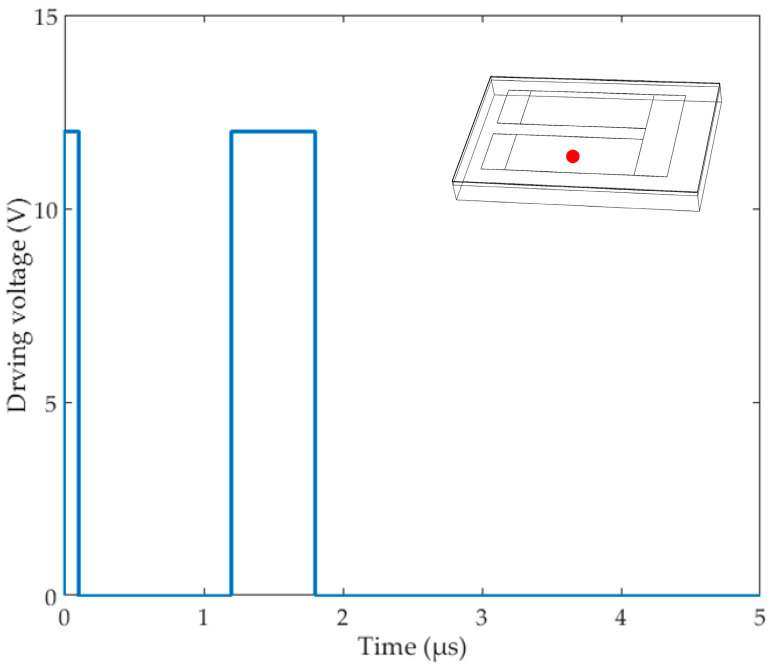
The driving signal of the heating resistors and the key point on the surface.

As shown in Figure 4, we chose a key point of the Si_3_N_4_ film to research: the center of one TaN film. Then, according to the temperature change at this point, the appropriate voltage was selected to drive the thermal bubble inkjet chip. Finally, we needed to set the working time of the heating resistors according to the previous test results found by our team, so the heating time here was 1.8 μs. The TaN film worked for 0.1 μs firstly, then worked from 1.2 μs to 1.8 μs. This is a classic heating method of thermal inkjet printing: the preheating time was short, and the objective was to preheat the temperature of the liquid in the chamber. Since the thermal transfer of the liquid was carried out by the movement of its internal molecules [28], the performance of thermal transfer was not as good as that of the solid, so we set a buffer time of 1.1 μs, and then a main heating time of 0.6 μs, thus forming a complete driving pulse signal.

**Figure 5:**
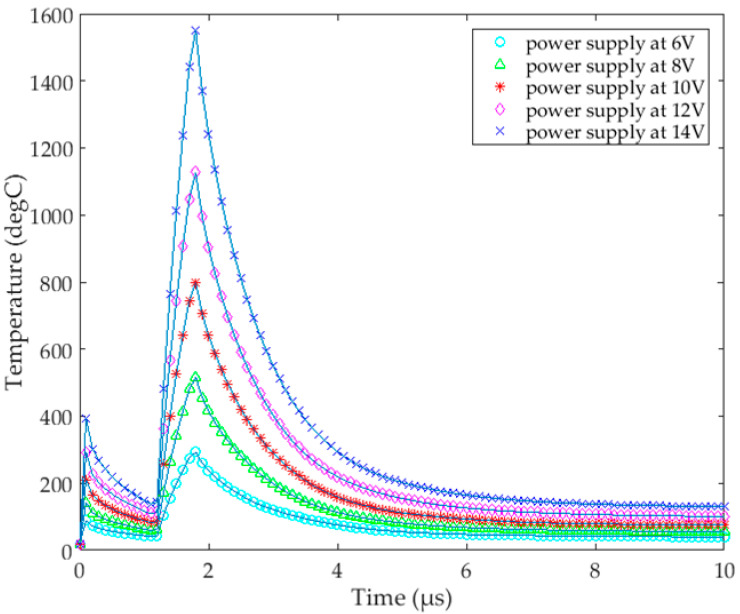
The temperature changes of the selected key point at different times at the different driving voltages.

**Figure 6:**
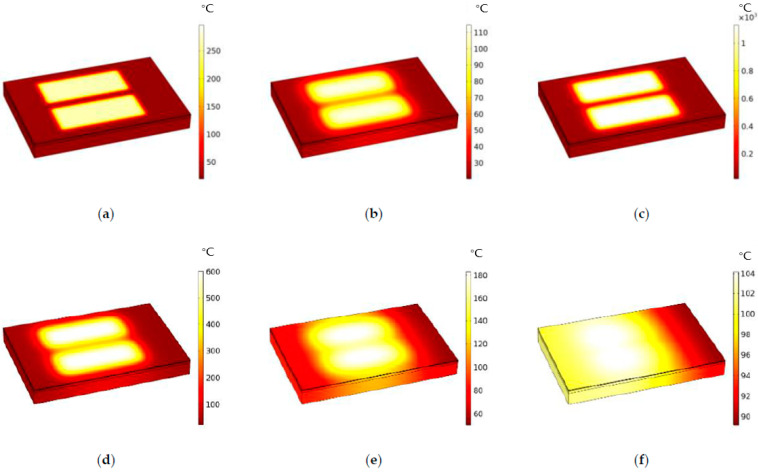
The simulation results for the TaN chopsticks-shaped heating resistors under a driving voltage of 12 V: (**a**) temperature distribution of the heating resistors at 0.1 μs; (**b**) temperature distribution of the heating resistors at 1.2 μs; (**c**) temperature distribution of the heating resistors at 1.8 μs; (**d**) temperature distribution of the heating resistors at 2.5 μs; (**e**) temperature distribution of the heating resistors at 4.5 μs; (**f**) temperature distribution of the heating resistors at 9 μs.

After the parameters were set, we used the finite element software to simulate the 3D model. As shown in Figure 5, the peak temperatures of the point appeared at 0.1 μs and 1.8 μs. When the voltage increased, the temperature of the heating resistors’ surfaces also rose. At a voltage of 14 V, the maximum temperature at 0.1 μs was about 400 °C, and the highest temperature was around 1550 °C at 1.8 μs. In the overall results, the surface of the heating resistors heated up faster, which could quickly transfer the thermal from the TaN film to the surface. When the TaN film stopped working, the temperature of the surface decayed rapidly. At about 9 μs, the temperature on surface dropped to about 200 °C. Based on practical experience, we finally decided to use 12 V, because under 14 V, the temperature at 0.1 μs was too high, which could cause the liquid to directly vaporize and eject out of the nozzle. The highest temperature at 1.8 μs could cause the rupture of Si_3_N_4_ film, because the temperature of the TaN film increased instantly, while the temperature of the Si_3_N_4_ surface could not be heated evenly in the short time. The central region would have a high temperature, forming a temperature difference with the edge region. Due to the thermal expansion and uneven heating of the Si_3_N_4_ film, thermal stress was generated, which caused the surface to rupture. Therefore, we chose a driving voltage condition of 12 V. Figure 6 shows the simulation results of the TaN chopsticks-shape heating resistors at 0.1 μs, 1.2 μs, 1.8 μs, 2.5 μs, 4.5 μs, and 9 μs.

When the driving voltage was applied, the TaN film heated up rapidly. Since the weak coefficient of thermal conductivity of SiO_2_ is about 1.4(W/(m·K)), it played a certain insulation role, so the bare silicon at the bottom and the Si_3_N_4_ film at the top were buffered. We set the preheating time as 0–0.1 μs, and it was found that at 0.1 μs, as shown in Figure 6a, the maximum temperature of the surface reached about 300 °C. As the preheating ended, as shown in Figure 6b, the central area of the Si_3_N_4_ began to cool down, to about 250 °C at 1.2 μs, while the heat began to diffuse into the surrounding area. As shown in Figure 6c, when the heating time was 1.8 μs, the maximum temperature of the central area reached about 1100 °C. Then, over time, the temperature began to homogenize across the surface. As shown in Figure 6e, the temperature distribution on the surface was between 80 and 200° at 4.5 μs.

We mentioned in [29] that the vaporization temperature of ink liquid was about 300 °C, and the maximum surface temperature of heating resistors could reach at 1100 °C. Therefore, we also needed to research a simulation of the thermal transfer between the solid and fluid.

**Figure 7:**
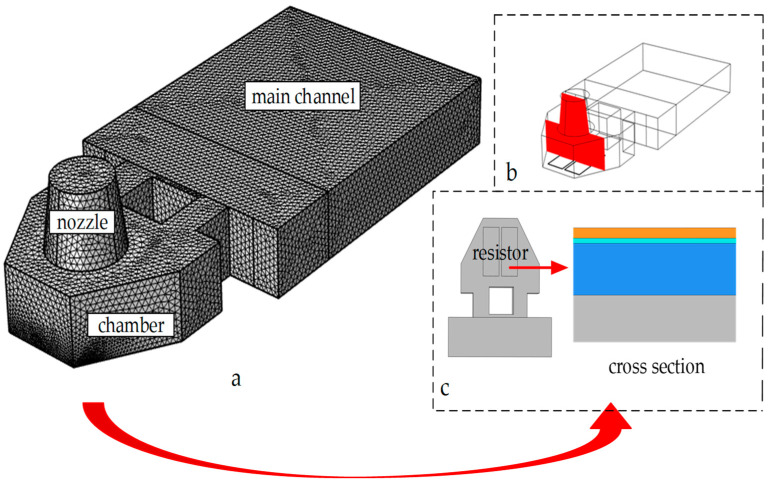
The establishment and meshing of thermal inkjet printing model: (**a**) the meshing of the thermal inkjet printing head; (**b**) a cross section in the chamber; (**c**) the heating resistors on the back of the printing head and a cross section of the resistors.

As shown in Figure 7, the thermal bubble inkjet printing head was divided into 2,077,374 grid cells, and the TaN chopsticks-shaped heating resistors were more densely divided due to their complex structural layers. We selected a cross section to research the state of thermal transfer in the chamber. The physical field of the 3D model was the thermal transfer between the solid and fluid. In addition, we also needed to research the viscous force of the fluid.

(6) $$\tau =\varepsilon \frac{dv}{dz}$$

where *τ* is the viscous force, *ε* is the viscosity coefficient, and *dv/dz* is the speed gradient in the normal direction of the plane. In the simulation, the ink viscosity was set to 10 mPa·s.

In thermal bubble inkjet printing, the heating resistors instantly reach a high temperature, which heats up the liquid in the chamber and nucleates the vapor bubble. During this time, the vapor pressure will push the fluid to eject from the nozzle at high speed. Due to the weak thermal conductivity of liquid, the time of thermal transfer between the solid and liquid is longer than that between solids. 

**Figure 8:**
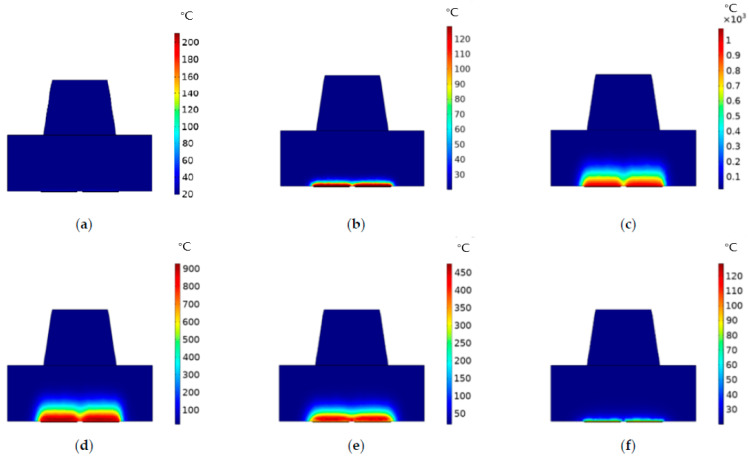
The temperature distribution of fluid in the chamber under a driving voltage of 12V: (**a**) temperature distribution of the fluid in the chamber at 0.1 μs; (**b**) temperature distribution of the fluid in the chamber at 1.2 μs; (**c**) temperature distribution of the fluid in the chamber at 1.8 μs; (**d**) temperature distribution of the fluid in the chamber at 2.5 μs; (**e**) temperature distribution of the fluid in the chamber at 4.5 μs; (**f**) temperature distribution of the fluid in the chamber at 9 μs.

As shown in Figure 8a, under the conditions of a 12V driving voltage and 0.1 μs, the state of thermal transfer in the chamber was not obvious, and the temperature at the bottom of the cross section was about 60 °C. The heating resistors stopped working between 0.1 and 1.1 μs; due to the existence of preheating, the temperature on their surfaces still existed, and the temperature at the bottom of the chamber was transferred upward during this period. Therefore, when the heating resistors worked again at 1.2 μs, as shown in Figure 8b, the liquid temperature in the chamber was about 110 °C. As shown in Figure 8c, when the heating time reached 1.8 μs, the internal temperature of the bubble reached 600 °C. The temperature at the bottom of the chamber reached about 800 °C, and the heat was still transferring upward. Therefore, when the heating resistors stopped working, as shown in Figure 8d, the vapor bubble did not shrink significantly. When the time was 4.5 μs, as shown in Figure 8e, it could be clearly seen that the vapor bubble was obviously reduced. The internal temperature was maintained at about 350 °C, and the temperature was evenly distributed inside the vapor bubble. As shown in Figure 8f, when the time reached 9 μs, the vapor bubble finally disappeared, and the temperature at the bottom of the cross section was about 70 °C.

**Figure 9:**
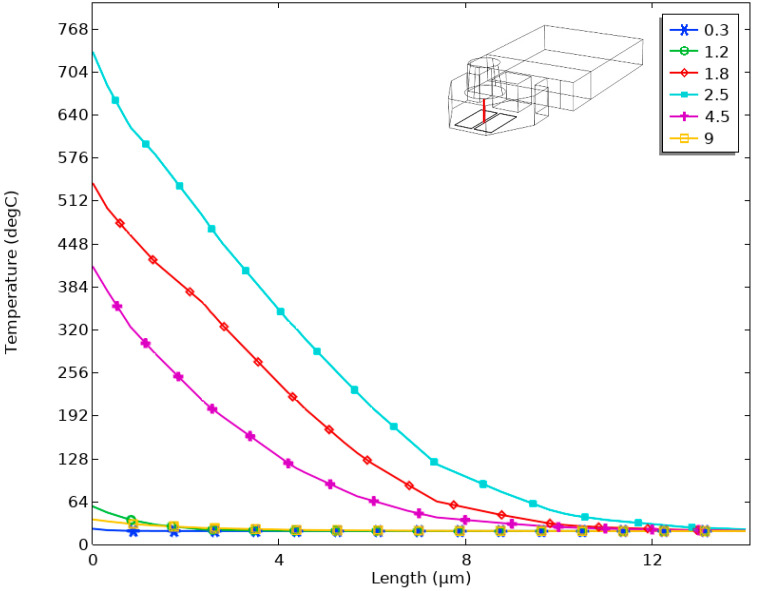
At different times, the temperature changed with the different heights of the straight line.

In order to research the change in temperature in the chamber more directly, we chose a red three-dimensional straight line as the object. In the upper-right corner of Figure 9, the line is located in the middle of the chopsticks-shaped heating resistors, perpendicular to the bottom of the chamber, at a height of 15 μm. The x-axis corresponds to the height of the straight line, and the y-axis corresponds to the temperature. When the time was 0.1 μs, 1.2 μs, 1.8 μs, 2.5 μs, 4.5 μs, and 9 μs, respectively, Figure 9 shows the temperature change that corresponded to the straight line at different heights. It was found that the temperature at the starting point of the straight line was the maximum. When the time was 2.5 μs, the temperature reached about 680 °C. When the height of the straight line was about 15μm, the corresponding temperature was about 30 °C. In fact, the evaporation temperature of the ink liquid could only be reached when the temperature was above 300 °C, so we estimated that the maximum height of the bubble was about 4 μm, and the corresponding time was 2.5 μs. However, when comparing the temperature distribution in Figure 6 with Figure 8, we found that the temperature in the straight line shown in Figure 9 did not reach the maximum temperature in the chamber shown in Figure 6. The reason was that the straight line we chose was in the middle of the cross section and did not directly contact the top surface of the heating resistors.

## 3. Experimental Results

### 3.1. Device Fabrication

**Figure 10:**
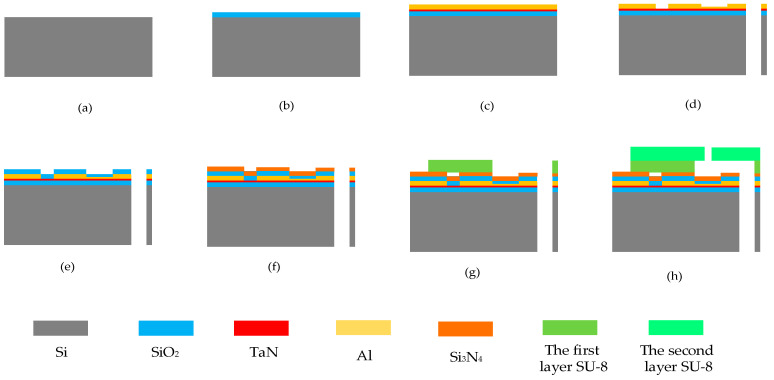
Manufacturing process of the thermal printing chip. (**a**) Using a bare silicon wafer as the substrate. (**b**) Using the first layer of SiO_2_ as the thin layer. (**c**) Using the TaN and Al to form the heating resistors. (**d**) Etching the chip using the MEMS process. (**e**) Using the second layer of SiO_2_ as the thin layer. (**f**) Using the Si3N4 as the thin layer. (**g**) Using the first layer of SU-8 photoresist to form the chamber. (**h**) Using the second layer of SU-8 photoresist to form the nozzle.

In this study, the School of Optoelectronic Engineering at Xi’an Technological University was commissioned to use micro-electro-mechanical system (MEMS) micromachining technology to manufacture the thermal bubble inkjet chips. As shown in Figure 10, the manufacturing process of the chip was basically the same as for the structure in [29], except for two improvements. The first improvement was that we added a layer of SiO_2_ film between the TaN film and the bare silicon to isolate the high temperature, using a thickness of 1μm. The second improvement was that we added a layer of SiO_2_ film on the surface of the TaN with a thickness of 0.1 μm. As a buffer layer, it avoided the thermal transfer from the TaN film to the Si_3_N_4_ film directly, resulting in its rupture. As compared with Figure 2, we integrated the fabrication of the chamber and nozzle into thermal inkjet printing chip, which mainly used the SU-8 photoresist and disposed it on the heating resistors. The first layer of the SU-8 photoresist was used to form the chamber with a height of 20 μm. The second layer of the SU-8 photoresist was used to form the nozzle with a height of 20 μm as well. As mentioned in [29], the reason for the same thickness of two layers of the SU-8 photoresist is mainly to save costs.

**Figure 11:**
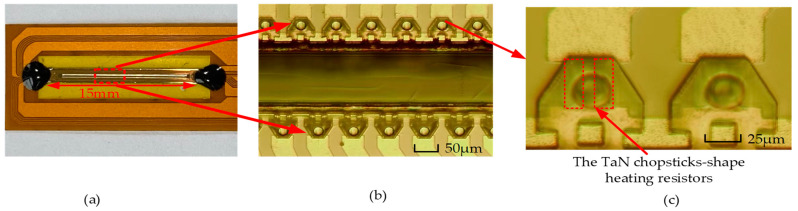
The structure of thermal bubble inkjet chip: (**a**) the overall preview of the thermal bubble inkjet chip; (**b**) the internal structure of the thermal bubble inkjet chip; (**c**) the positions of the TaN chopsticks-shaped heating resistors.

As shown in Figure 11a, the length of the thermal bubble inkjet chip designed by our team was about 15 mm, and the inkjet chip contained 360 inkjet printing heads. As shown in Figure 11b, the inkjet printing heads inside the chip were evenly distributed on both sides of the main channel. It should be noted that the positions of the upper and lower sides of the inkjet printing heads were staggered, and the distance of the stagger was about 50 μm. In the process of inkjet printing, the ink liquid in the main channel flowed to the chamber after each inkjet was completed. If the positions of the inkjet printing heads on the upper and lower sides of the main channel were not staggered, it would affect the filling speed of the ink liquid. In Figure 11c, the positions of the TaN chopsticks-shapes heating resistors are shown by the dotted red rectangles. Since the inkjet chips were made using MEMS micromachining technology, the nozzle and chamber on the top layer were fabricated with the SU-8 photoresist, so they could be seen firstly under the optical microscope, while the positions of heating resistors were covered by the SU-8 photoresist, so they were not obvious. In fact, if we looked closely at the inkjet printing head on the right side shown in Figure 11c, we could still see the positions of the heating resistors.

During the inkjet debugging, our team found an unexpected problem. During the high-frequency continuous inkjet printing, the ink in the chamber could not be ejected out normally. We carefully checked the internal circuit of the inkjet chip, and no damage was found. If the internal structure and circuit of the chip were intact, then the problem lay in the MEMS process. We checked the design of the main channel to make sure it would fill the chamber in time. Finally, we found that the fatal problem was the interface between the back of the chip and the cartridge, so the ink would not flow from the cartridge to the chip in time. Since the material on the back of the chip was Si, our team considered covering it with a layer of SiO_2_ film. 

**Figure 12:**
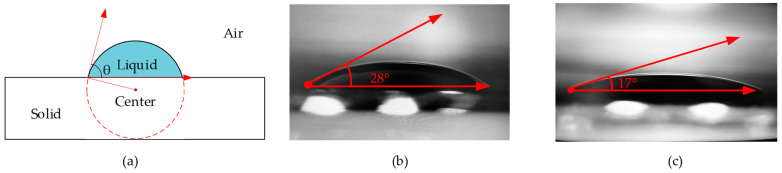
Measurement of the contact angle. (**a**) The principle of contact-angle measurement; (**b**) measuring the contact angle of water-based ink on Sithe ink products by the company of Asdun, Shenzhen, China); (**c**) measuring the contact angle of water-based ink on SiO_2_.

Since the SiO_2_ had a higher hydrophilicity than the Si, as shown in Figure 12, the contact angle of the water-based ink on the Si was about 28 °C, and that of the ink on the SiO_2_ was about 17 °C. This showed that SiO_2_ had a better hydrophilicity than Si, so the ink could be supplemented inside the chip in time after it was connected to the cartridge [30].

### 3.2. Thermal Bubble Inkjet Printing Test

In our previous work in [29], we measured the ink droplet injection volume and verified the printing frequency of the inkjet printing head. The inkjet test was verified on only one inkjet printing head previously, while the inkjet chip designed by our team had up to 10 inkjet printing heads working simultaneously in the full inkjet printing. This meant that when the inkjet chip was working continuously at a high frequency, there should have been 10 ink-mark lines. Therefore, we needed to test the working condition of the inkjet chip under the 12 V driving voltage. If the inkjet printing heads inside the chip worked properly and the ink-mark lines were sufficiently clear, this showed that the results were consistent with the previous simulations.

**Figure 13:**
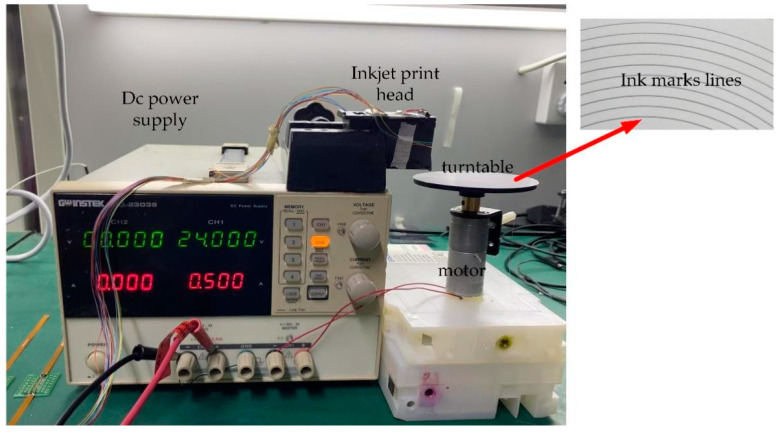
Establishment of experimental environment.

As shown in Figure 13, we used a power supply to drive an electric motor. A turntable was installed on the spindle of the motor, which was about 1 cm away from the inkjet printing chip. The reason was that after the ink droplets were ejected from the nozzle, due to the fast speed of the ejection, the air resistance was large, and the volume of the ink droplets was small, which was vulnerable to influence by the micro air flow. Thus, if the distance between the printed paper and the inkjet chip was excessive, the phenomenon of ink deviation easily occurred, which affected the quality of the inkjet printing. The turntable was covered with glossy photo paper because the ink droplets would permeate and diffuse on traditional wood paper, making later observation difficult. The electric motor speed was 500 revolutions per minute (rpm), and the driving voltage was 24 V–0.5 A. Under full power, the period of the motor $T_{i}$ was about $\frac{3}{25}$ s per round. The reason we chose the high-speed motor was that the time interval between the high-frequency inkjet was too short. In order to research the shape of the ink droplets, the high-speed motor had to be used for matching. When the speed was slow, the ink droplets would overlap each other, which was not convenient to later research. In the upper-right corner of Figure 13, we show the actual ink-mark lines in advance. A total of 10 ink-mark lines can be clearly seen, which indicated that the inkjet printing chip could control 10 inkjet printing heads at the same time.

In addition, as shown the ink-mark lines in Figure 13, the printing paths of the inkjet chip on the turntable were curved lines, not straight, which did not affect the tests in our research. Because the period of the inkjet is was short and the frequency was fast, according to Newton’s calculus theory, the curve could be divided into the accumulation of countless straight lines, so the ink-mark lines under the microscope should have looked like straight lines.

**Figure 14:**
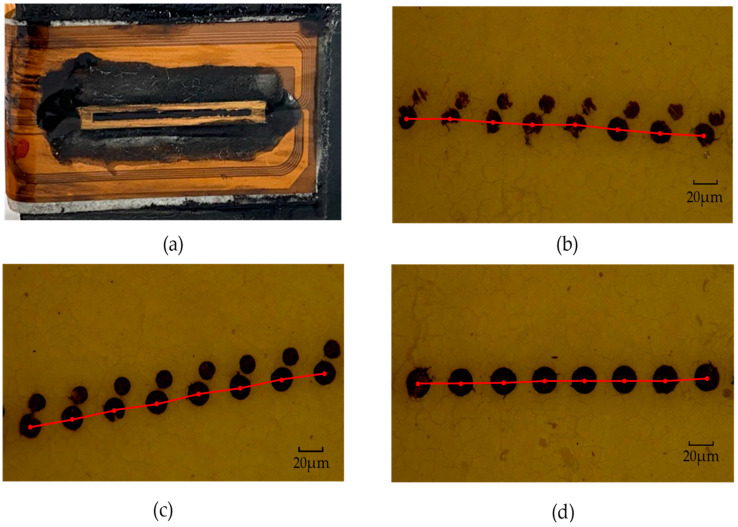
The ink marks lines of inkjet printing under the different driving voltages as observed under an optical microscope (made by Shenzhen Aosvi Optical Instrument Co., Ltd., Shenzhen, China, and with ink produced by the company of Asdun, Shenzhen, China). (**a**) When the driving voltage was 6V, the inkjet chip could not work normally, and an ink blockage occurred at this time. (**b**) The printing path of the inkjet chip when the driving voltage was 8 V. (**c**) The printing path of inkjet chip when the driving voltage was 10 V. (**d**) The printing path of inkjet chip when the driving voltage was 12 V.

As shown in Figure 14a, when the driving voltage was 6 V, the inkjet chip could not work properly. However, as shown in Figure 6, when the voltage was 6 V, the highest temperature of the heating resistors was about 300 °C, and this temperature was too low to cause most of the liquid in the chamber to vaporize instantly in a short period, while there was lower ink-droplet overflow from the nozzle. Due to the surface tension and the viscous force between the inks [31], the ink droplets eventually accumulated around the nozzle. When the time of inkjet printing was increased, the accumulation of ink liquid around the nozzle also increased, which prevented the subsequent ink droplets from being ejected from the nozzle properly, and eventually caused the nozzle blockage. It should be added that as shown in Figure 14a, there was a black material around the chip, which was the ultraviolet (UV) glue. When we connected the chip to the cartridge, in order to ensure that the ink flowed from the cartridge to the chip completely, we used a layer of UV glue to cover on the joint and illuminate it with ultraviolet light to cause it to solidify.

As shown in Figure 14b,c, when the driving voltage was 8 V and 10 V, respectively, the inkjet chip ejected two droplets each time, one large and one small—these were the satellite droplets. The formation of the satellite droplets was mainly related to the viscous force and surface tension between the fluids, which seriously affected the printing quality [32]. As shown in Figure 14d, when the driving voltage was 12 V, the size and shape of the ink droplets were almost the same, and the distance between adjacent ink droplets was almost the same. This phenomenon showed that the inkjet print head could eject normally at a frequency of 40 kHz.

In order to further research the inkjet performance of the droplets, we marked the centers of the ink droplets, as shown in Figure 14. At a printing frequency of 40 kHz, if the distance between two adjacent centers remained constant, that meant the inkjet print head could work properly. As mentioned earlier, Newton’s calculus theory divides a curve into an accumulation of numerous straight lines. Under the optical microscope, we could observe that the ink-mark lines were close to a straight line. The formula for the distance $L_{i}$ between two adjacent centers is:

(7) $$L_{i}=V_{i}t_{i}$$

where *V* represents the speed and *t* is the interval time of printing. Because the printing frequency was 40 kHz, the time $t_{i}$ was 25 μs. The speed $V_{i}$ can be expressed by the linear velocity of the turntable; the formula is:

(8) $$V_{i}=\frac{2\pi r}{T_{i}}$$

where *r* is the distance from the ink-mark line to the center of the turntable, and $T_{i}$ is the rotation period of the motor ($T_{i}=\frac{3}{25}$ s per round). As shown in Figure 14, the distance *r* between the 8 V, 10 V, and 12 V ink-mark lines and the center of the turntable was about 2.65 cm. According to Formula (4), the theoretical distance *r* between two droplets was about 34.67 μm. 

**Figure 15:**
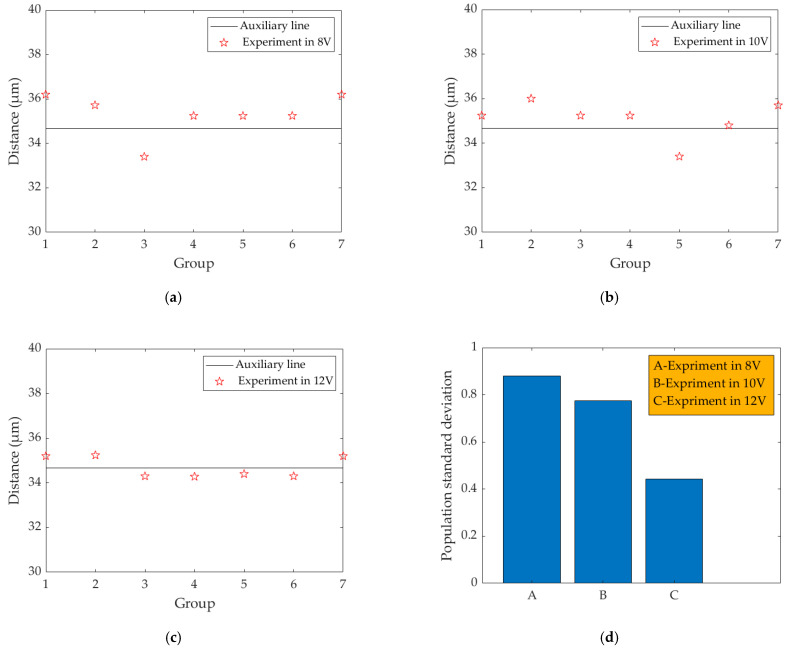
The distance between two adjacent centers at different voltages: (**a**) at 8 V; (**b**) at 10 V; (**c**) at 12 V. (**d**) The population standard deviation of the distance between adjacent droplets under the different driving voltages.

As shown in Figure 15a,b, when the voltage was 8 V and 10 V, respectively, the distance between the two adjacent ink droplets deviated greatly from the theoretical value. As shown in Figure 15c, the distance between two adjacent ink droplets was almost consistent with the theoretical value, and part of the error was caused by the selection of the center point and the inevitable error in the experiment. As shown in Figure 15d, when the driving voltage was 8 V, 10 V, and 12 V respectively, the population standard deviation of the distance between adjacent ink droplets was 0.879 (8 V), 0.775 (10 V), and 0.447 (12 V), respectively. It was clear that when the driving voltage was 12 V, the population standard deviation was the smallest, and the inkjet performance was the best.

**Figure 16:**
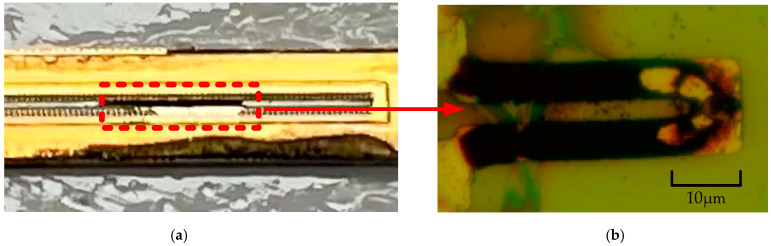
When the driving voltage was 14 V, the inkjet chip is burned. (**a**) Under the microscope, we used tweezers to scrape the SU-8 photoresist off the surface of the chip, and the nozzle and chamber were removed to facilitate the observation of heating resistors. (**b**) The heating resistors were burnt out due to the excessive voltage.

**Figure 17:**
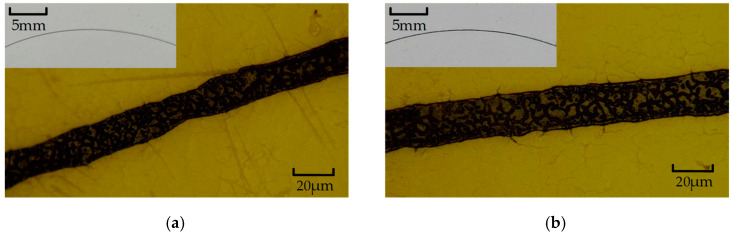
Reducing the speed of motor, using the different voltages to drive the inkjet chip and observe the ink-mark lines. (**a**) The printing path of the inkjet chip when the driving voltage was 10 V. (**b**) The printing path of the inkjet chip when the driving voltage is 12 V.

As shown in Figure 16, when the driving voltage was 14 V, the inkjet chip was broken. After we removed the chip from the cartridge, we could not directly observe the surface of heating resistors. As shown in Figure 11c, the heating resistors were covered with two layers of SU-8 photoresist, so we used tweezers to scrape them and remove the nozzle and chamber on the surface. As shown in Figure 16, the surface of the heating resistors was burned. At this time, because the power of the TaN heating resistors was excessive, the Si_3_N_4_ film was heated up sharply in a short time, so its surface temperature distribution and thermal stress were excessive, leading to the surface breaking and falling off. The black section shown in Figure 16b was the surface of TaN heating resistors. We can see clearly that the TaN film was almost completely burned. In order to research the working performance of the inkjet chip, we previously used a high-speed motor to observe the volume of the ink droplets. As shown in Figure 17, when the driving voltage was 10 V and 12 V, the volume of the ink droplets was relatively large. In fact, the speed of the motor inside the printer was slow, so it was necessary to consider whether the ink was too thick and affected the quality of the print. Thus, we reduced the driving voltage of the motor to 12 V to observe the ink-mark lines from the chip. 

As shown in Figure 17a, as the speed of motor decreased and the inkjet frequency remained constant, there was an overlap of the adjacent ink droplets, eventually forming a straight line. The ink-mark line shown in Figure 17a was not full enough, and there was insufficient ink in some parts. The upper-left corner of the Figure 17 shows the ink-mark line on the turntable. It can be obviously seen that the actual ink-mark line was not clear due to the insufficient inkjet amount. As shown in Figure 17b, the width of the ink-mark line was evenly distributed, and the actual ink-mark line in the upper left of the figure was clear. Therefore, we finally chose 12 V to drive the inkjet chip.

## 4. Conclusions

In this study, we proposed TaN chopsticks-shaped heating resistors. The thickness of the TaN film was about 0.05 μm, and the thickness of the Si_3_N_4_ film was about 0.2 μm. A layer of SiO_2_ film was added between the TaN and Si_3_N_4_ films as a buffer layer, which prevented the surface of the latter from being destroyed by the excessive thermal stress in the short time. The electric thermal measurement model was established using finite element simulation software. Because the vaporization temperature of the ink liquid had to reach more than 300 °C, and an excessive voltage would burn out the resistors, we finally chose a driving voltage of 12 V. The working time of the heating resistors was 1.8 μs, of which the preheating time was 0.1 μs and the main heating time was 0.6 μs. The surface temperature of heating resistors could reach about 1100 °C. Then, the temperature distribution of the liquid in the chamber was researched by the solid–liquid heating-transfer model. Since the coefficient of thermal conductivity of the liquid was smaller than that of solid, there was a time delay when heat was transferred from the heating resistors to the liquid. The vapor bubble expanded to the maximum at 2.5 μs, and finally disappears at 9 μs. At 2.5 μs, the maximum temperature inside the bubble was about 700 °C.

Next, we used MEMS micromachining technology to fabricate the inkjet printing chip. During the inkjet debugging, the ink-supply efficiency between the cartridge and the chip was poor. We solved this problem by covering a layer of SiO_2_ film on the back of the chip. Then, the respective driving voltages of 6 V, 8 V, 10 V, 12 V, and 14 V were applied to the inkjet chip to control 10 inkjet printing heads. We found that when the driving voltage was 6 V, the inkjet printing heads could not work normally, and ink blockage appeared at the nozzle. When the driving voltage was 14 V, the heating resistors were burned. When the driving voltage was 8 V and 10 V, the ink was not full, and the ink-mark lines were not clear enough. Finally, we determined a driving voltage for the TaN chopsticks-shaped heating resistors of 12 V and a printing frequency of 40 kHz. In the existing mainstream thermal bubble inkjet printing chip, the inkjet printing frequencies for Canon, HP, and Epson are mostly at 15 kHz, while the inkjet chip designed in this paper had a printing frequency as high as 40 kHz. The inkjet chip in our study is not limited to daily office printing, but also provides a new equipment option for 3D printing; cell printing; and plant, vegetable, and fruit printing.

## Figures and Tables

**Table 1:** The design parameters of the heating resistors.

Structure Parameters	Value (μm)
Si	60 × 40 × 5
The first layer of SiO_2_	60 × 40 × 1
TaN	32 × 13 × 0.05
Al	6 × 13 × 0.05; 8 × 13 × 0.05,
The second layer of SiO_2_	60 × 40 × 0.1
Si_3_N_4_	60 × 40 × 0.2

**Table 2:** The main parameters of the materials during the simulation, t.

Materials	Constant Pressure Heat Capacity (J/(kg·k))	Density (kg/m^3^)	Thermal Conductivity (w/(m·k))	Conductivity (S/m)
Silicon	700	2329	130	10^−12^
SiO_2_	730	2200	1.4	0
TaN	522	14,506	9.54	0.5 × 10^6^
Si_3_N_4_	700	3100	20	0
Al	900	2700	238	3.774 × 10^7^

## Data Availability

The data presented in this study are available from the corresponding author upon reasonable request.
